# Southern Surgical and Gynecological Association

**Published:** 1900-12

**Authors:** 


### *SOCIETY REPORTS.*

---

### SOUTHERN SURGICAL AND GYNECOLOGICAL ASSOCIATION.

The Southern Surgical and Gynecological Association convened at Atlanta November 13, 14, 15.

After an address of welcome by Governor A. D. Candler, the association was called to order by the President, Dr. A. M. Cartledge, of Louisville, Ky.

Dr. W. L. Rodman read a paper on the subject of Medullary Narcosis. This method will not supersede ether and chloroform in operations below the diaphragm. It is to be held in reserve in cases where these agents are contraindicated, as in persons suffering from bronchial, pulmonary and renal disease, particularly with fatty or dilated hearts or cardiac diseases in general. It is indicated in old people, and, perhaps, in drunkards. Any of the lumbar spaces may be selected for the injection—the fifth, that between the last lumbar and first sacral vertebræ is, in some respects, the easiest. The dangers of simple puncture do not appear to be great. Secondary changes leading to chronic disease of the chord have not been shown. He recommended half the amount of the two per cent. solution of cocaine, suggested by Tuffier, and believes 18 to 13 minims sufficient. The aim is to secure complete anesthesia with the minimal amount of the drug. Blindfolding the patient and plugging the ears with cotton are desirable to prevent psychic pain.

In the discussion which followed Dr. McMurtry, of Louisville, agreed with the reader that the method would not take the place of the established methods, and that its field of usefulness would be confined to the class of cases outlined.

Dr. W. G. McDonald, of Albany, mentioned a case of an alcoholic to be operated upon for hemorrhoids, in whom the injection was very nearly fatal.

Dr. Cartledge favored the method, and had used it with success in eight cases.

Remarks were made by Drs. Mallett of New York, McMonagle of San Francisco, and Seneca D. Powell of New York, and were, in the main, commendatory of the method.

Removal of Pelvic Inflammatory Masses by the Abdomen after Bisection of the Uterus, was the title of a paper by Dr. Howard A. Kelly of Baltimore, in which he detailed the steps of the operation and its advantages. The discussion was participated in by Drs. McDonald, G. H. Engelman of Boston, W. E. B. Davis of Birmingham, and J. W. Bovee of Washington, D. C.

Vesicovaginal Fistula was considered by Dr. M. G. McGannon of Nashville. In operating he followed the principle of Mackenrodt, and found it satisfactory in the six cases operated upon by him in the last two years.

Osteo-fibroma of the Uterus. Dr. George Ben Johnston of Richmond, detailed an instance of this rare condition which occurred in his practice, and which was successfully removed.

Appendicitis in the Female—Dr. F. W. McRae. The author believed that the great disparity in the statistics of the relative frequency of the disease in male and female, put as 80 per cent. in favor of the former, is due to mistakes in diagnosis. Sufficient stress has not been laid upon the fact that appendicitis in women usually occurs at or about the menstrual period, a circumstance tending to favor mistakes in diagnosis. Interesting cases illustrating the points at issue were cited by Drs. J. B. S. Holmes of Atlanta, Hal C. Wyman of Detroit, Kelly of Baltimore, McMurtry of Louisville, W. P. Nicolson of Atlanta, and George Ben Johnston of Richmond. Dr. Johnston believed that the fulminating form of appendicitis is less frequent in women than in men. He noted the frequent association of the disease with floating kidney.

Drainage in Abdominal Surgery. In his paper, Dr. J. W. Long of Salisbury, N. C., said that drainage was designed to carry away septic matters, blood and other fluids from the peritoneal cavity; to promote adhesions for the protection of weak spots, and to permit a more accurate knowledge of the conditions present. Objections to drainage are that it is deceptive. Cases drained do no better than those not so treated; drainage is neither scientific nor workmanlike.

Dr. Manning Simmons of Charleston, S. C., agreed in general with the speaker, but he believed drainage to be applicable in diffuse suppurative conditions of the pelvic and abdominal cavities.

Dr. Kelly remarked that drainage was too frequently used. He would drain cases of local sepsis where the entire septic area had not been removed.

Dr. W. E. B. Davis referred to the use of drainage in general septic peritonitis, and commended it in connection with peroxide of hydrogen flushing and normal salt solution given under the skin.

Dr. Beverly McMonagle had found a small drain necessary after operations on the gall-bladder and gall ducts.

Atresia of the Vagina. Dr. George H. Noble, of Atlanta, described and illustrated a flap operation for the relief of this condition, which had proven successful in his hands.

Technical Improvements in Surgery of the Stomach for Carcinoma.

In this paper, Dr. W. G. McDonald, of Albany, gave the indications for operation, the various technical methods and the instruments used, the manner of suturing to secure anastimosis, preferring a Murphy button for secondary anastimosis.

The mortality attending the surgery of the stomach has steadily declined under technical improvement, the average being, according to Mayo Robson, about 30 per cent.

Menstrual Condition of the Average Girl in Average Health. Dr. Geo. J. Engelman, of Boston, presented the paper. Based upon a study of the records of 4,873 cases from high and normal schools, colleges and department stores; the girls being between fifteen and twenty-six years of age, and in general good health. The following facts were observed as to menstruation: Regularity in 50 per cent. of the cases only; recurrence every 28 days in 30 per cent., varying most frequently from 26 to 42, 45 per cent. being over 28. The average duration was 4.6 days; from 66 to 70 suffer more or less. Some few are habitually incapacitated from work and 30 per cent. occasionally. The performance of this function in the average American girl in good health is, therefore, not ideal, and the study and correction of the evil lies with physicians and educators.

Operation for Marked Prolapse of the Rectum in Women. Dr. J. Wesley Bovée, of Washington, described under this title an operation for the relief of the condition. It consisted in suturing the rectum to the posterior cul-de-sac and posterior portion of the uterus, which was attached to the abdominal wall.

Carbolic Acid in Surgery. Dr. Seneca D. Powell read this paper and referred to his frequent use of the agent in the treatment of microbic disease. The cases embraced all phases of bone diseases and infections. The action of the acid could be controlled by alcohol, its antidote.

Excision and Irreducible Dislocations. Dr. Willis F. Westmoreland narrated two cases of irreducible dislocation in which excision of the head of the humerus was done with a successful result.

A Plea for a Better Appreciation of the Limitations of Operative Work. This was the title of the President's address, delivered by Dr. A. M. Cartledge. He entered a plea, after historical allusion to diagnostic methods in the past, for more careful diagnoses with an especial view to detecting attending visceral disease in order to become more expert prognosticians. There are two phases of surgical practice alone, the careful observance of which would reduce the mortality of surgical operations so low as to cause us to believe that exactness had been reached. He referred to the greater care in the detection of kidney lesions and instituting measures to correct this frequent cause of unfortunate operative terminations, and the still prevalent practice of operating upon hopeless cases of cancer. Surgical limitation in operations upon the pelvic and abdominal cavities is most often exceeded and the mortality unnecessarily increased in operations for the following diseases: General septic peritonitis, extensive carcinoma of the ovaries, uterus and intestines, operations upon the gall passages, in long-continued and profoundly cholemic patients, without adequate preparation. He protested against operation in these affections, believing it to be unwarrantable.

Excision of the External Carotid in Malignant Disease of the Face. Dr. William Perrin Nicholson. (See elsewhere this journal.)

Autointoxication for Renal Insufficiency with or without Disease of the Kidneys. Dr. James T. Jelks of Hot Springs, Ark. Renal insufficiency is often overlooked as the basis of many affections. Faulty elimination may be ascertained by a methodical examination of the twenty-four-hour urinary output. Such patients will be benefited by remedies such as squills, phosphate of soda, Vichy water, dietary restrictions, baths, and hot water taken internally in quantities amounting to a half to one gallon daily.

Dr. George Brown of Birmingham, made a further report on a case of litholopaxy and one of vesico-rectal fistula, previously presented to the Association.

Dr. J. A. Goggans reported some surgical cases.

Life-Saving Measures in Obstetric Work. Dr. R. R. Kime of Atlanta.

Dr. Kime said that aside from larger operative procedures, the life-saving measures saline infusions, medicinal remedies, serotheropy, hydrotherapy and drainage were the most important. Saline infusions or intravenous injections were of prime importance in placenta previa, not only to save life, but to lessen susceptibility to infection and hasten recovery.

Pseudo-Membraneous Enteritis and Abdominal Surgery. Dr. Frank A. Glasgow, of St. Louis, called attention to this disease and its relations to appendicitis.

Solid Ovarian Tumor. Dr. John G. Earnest, of Atlanta, reported a case of this growth. It was fibroid in character, measured twenty centimeters in length and about fourteen in breadth at the widest point. The patient from whom the growth was removed made an uninterrupted recovery.

Histogenesis of Ovarian Dermoids. W. D. Haggard, Jr., of Nashville, presented an abstract of a paper on this subject.

Dermoids were akin to teratoma; they contain all the blastodermic elements but are ovulogenous, and are not inclusions of ectoderm, as are dermoids elsewhere.

Papers were also presented by Dr. Howard A. Kelly on the Removal of Cystic Gall-stones; by Dr. Michael Hoke, on Osteo-Arthritis (see elsewhere in this journal); by Dr. W. F. Parham, of New Orleans, on Epi- and Hypospadias, with Special Reference to Treatment; by Dr. W. A. Quinn, of Henderson, Ky., on Retroflex Incarceration of the Gravid Uterus.

Officers elected for 1901: President, Dr. Manning Simmons of Charleston, S. C.; vice-presidents, George H. Noble of Atlanta, L. C. Boscher of Richmond; secretary, W. D. Haggard of Nashville; treasurer, F. W. McRae of Atlanta. Richmond, Va., was selected as the next place of meeting, third Tuesday in November, 1901.

While here the members of the Association were tendered a reception at the Kimball House, and were pleasantly entertained in other ways.

